# The Diagnosis, Management, and Outcomes of Gradenigo Syndrome in Children: A Scoping Review of the Literature

**DOI:** 10.3390/diagnostics15172193

**Published:** 2025-08-29

**Authors:** Charles Schmit, Felix Keller, Timo Gottfried, Roland Hartl, Lea Stecher, Andrea Tröger, Matthias Santer, Veronika Innerhofer, Avneet Radhawa, Joachim Schmutzhard, Benedikt Hofauer, Annette Runge

**Affiliations:** 1 Department of Otorhinolaryngology-Head and Neck Surgery, Medical University of Innsbruck, 6020 Innsbruck, Austria; charles.schmit@i-med.ac.at (C.S.); roland.hartl@tirol-kliniken.at (R.H.); lea.stecher@tirol-kliniken.at (L.S.); andrea.troeger@i-med.ac.at (A.T.); matthias.santer@i-med.ac.at (M.S.); veronika.innerhofer@tirol-kliniken.at (V.I.); joachim.schmutzhard@i-med.ac.at (J.S.); benedikt-gabriel.hofauer@i-med.ac.at (B.H.); annette.runge@tirol-kliniken.at (A.R.); 2 Department of Internal Medicine IV (Nephrology and Hypertension), Medical University of Innsbruck, 6020 Innsbruck, Austria; 3 Department of Otorhinolaryngology, Head and Neck Surgery, New York Presbyterian Hospital, New York, NY 10032, USA; mkx9001@nyp.org

**Keywords:** Gradenigo syndrome, petrous apicitis, acute otitis media, abducens nerve palsy, suppurative otitis media, unilateral facial pain, complications otitis media

## Abstract

**Purpose**: Gradenigo syndrome is a rare complication of acute otitis media (AOM) in children, characterized by suppurative otitis media, unilateral facial pain, and ipsilateral abducens nerve palsy. This review summarizes pediatric data on the presentation, diagnostics, treatment, and outcomes. **Methods**: A literature research was conducted using the terms “Gradenigo syndrome,” “petrous apicitis,” and “complications otitis media.” Pediatric cases were analyzed for demographics, symptoms, diagnostic findings, therapeutic strategies, and clinical outcomes. **Results**: Sixty-three articles described 65 patients (mean age: 8.0 years). The classic triad occurred in 22% of cases; 74% showed incomplete presentations. Imaging revealed petrous apex inflammation (84%) and petrous bone tip obliteration (49%). Antibiotics were administered in 88% of cases, most commonly third-generation cephalosporins. Surgery was performed in 72%, mainly myringotomy, tympanostomy tube insertion, and mastoidectomy; no direct petrous apex approaches were reported. Pathogens were identified in 41% of cases, most commonly Fusobacterium necrophorum. Clinical improvement occurred in 98%, with 75% achieving a complete resolution; complications were reported in 29%, including one fatality (2%). **Conclusions**: Given its variable presentation, comprehensive diagnostic imaging is essential for the diagnosis of Gradenigo syndrome. Early broad-spectrum antibiotic therapy is essential. Surgical intervention is required in severe cases. Long-term targeted antibiotic therapy may help prevent recurrence.

## 1. Introduction

Acute otitis media is one of the most common childhood illnesses, with more than half of all children experiencing at least one episode by the age of four [1,2,3,4,5,6]. The widespread use of antibiotics has significantly reduced severe complications in clinical practice [7,8,9,10,11]. However, when complications do occur, they can be life-threatening and may lead to long-term impairment [12,13,14]. Common complications include facial nerve palsy, labyrinthitis, cerebral abscesses, and mastoiditis—all of which require an immediate diagnosis and treatment [8,9,15,16,17]. One rare but notable complication of acute otitis media is Gradenigo syndrome.

First described in 1904 by Giuseppe Gradenigo, this syndrome is characterized by a triad of suppurative otitis media, unilateral facial pain in the distribution of the first two branches of the trigeminal nerve, and ipsilateral abducens nerve palsy, which results in horizontal double vision [18,19]. The progressive spread of infection to the petrous apex is pathognomonic of Gradenigo syndrome. The inflammatory impairment of the nearby fifth and sixth cranial nerve results in the classic symptoms [20]. The inflammatory involvement of nearby vessels, including the internal carotid artery and the cavernous sinus, is also possible [19,20].

Broad antibiotic therapy is the cornerstone of treatment for Gradenigo syndrome. If conservative management fails to produce clinical improvement, surgical intervention is required [19,21,22,23,24].

Although individual cases and small series have been described, available reports vary considerably in their descriptions of the clinical presentation, diagnostic work-up, and management.

This scoping review aims to address this gap by providing a comprehensive overview of clinical symptoms, diagnostic and imaging findings, therapeutic approaches, and post-treatment outcomes of Gradenigo syndrome in children.

## 2. Material and Methods

### 2.1. Search Strategy

**Figure 1:**
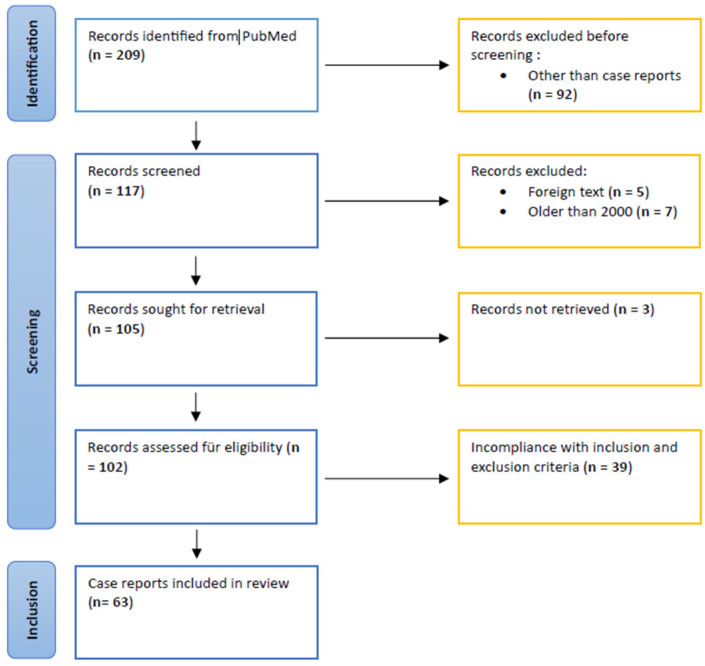
Identification of case reports.

This scoping review was not registered in PROSPERO, but the methodology follows established guidelines (e.g., PRISMA-ScR). A comprehensive literature review was performed using PubMed and Google Scholar. Case reports related to Gradenigo syndrome were identified using the following search terms: “Gradenigo syndrome”, “Gradenigo”, and “complications otitis media”. After the initial identification of potentially relevant articles, a total of 54 case reports were excluded from further investigation during the subsequent screening process. Figure 1 demonstrates the search strategy, inclusion process, and article selection. The query yielded 63 case reports.

### 2.2. Study Selection

The authors independently reviewed the titles, abstracts, and full-text articles of each search. Articles were initially screened by title and abstract. Articles with detailed information on symptoms, clinical and histopathologic findings, treatment, and outcomes were included and extracted.

Inclusion criteria were as follows:

- Diagnosed Gradenigo syndrome.
- Age ≤ 18 at the time of presentation.
- Full access to the case report.
- Case reports in English, German, or French.
  Exclusion criteria were as follows:
- Case reports in languages other than English, German, and French.
- Case reports lacking detailed information (symptoms, radiologic findings, treatment modality, and follow-up).
- Age > 18 years at the time of presentation.
- Case reports on non-human subjects.
- Case reports published before the year of 2000.

Discrepancies in analysis of articles among the reviewers were discussed until agreement was achieved.

### 2.3. Data Extraction

Age, sex, affected side, medical history, clinical presentation, laboratory parameters, radiological and microbiological findings, treatment modalities, and follow-up information were retrieved from the case report and recorded. Data extracted from each article were reported in counts and percentages or simple descriptive measures.

### 2.4. Statistical Analysis

Due to potential skewness and the moderate sample size, continuous variables were reported as the median with 1st and 3rd quartile (IQR). Discrete variables were presented as absolute and relative frequencies. All analyses were performed using R (version 4.4.2) [25].

## 3. Results

### 3.1. Search Results

The initial literature search across the databases yielded 209 articles. Among these, 117 were case reports, with 63 meeting the inclusion criteria. Of these, 61 articles were single-patient case reports [19,22,26,27,28,29,30,31,32,33,34,35,36,37,38,39,40,41,42,43,44,45,46,47,48,49,50,51,52,53,54,55,56,57,58,59,60,61,62,63,64,65,66,67,68,69,70,71,72,73,74,75,76,77,78,79,80,81,82,83,84], and two were case series [20,85]. In total, 65 pediatric patients were described in the included studies. Data were collected for each case on the following parameters: age, sex, affected side, time to presentation, prior antibiotic therapy, history of otitis media, clinical presentation, laboratory parameters, radiological findings, therapeutic procedures, microbiological results, and patient outcomes.

### 3.2. Patient Characteristics

This study included 65 pediatric patients, of whom 52% (33 patients) were female and 48% (30 patients) were male. The right side was affected in 52% of cases (34 patients), while the left side was involved in 48% (31 patients). The mean age of the patients was 8.0 years (IQR 5.0, 11.5). A history of recurrent otitis media was reported in 29% of cases (19 patients), while 43% (28 cases) experienced an acute episode of otitis media immediately prior to the initial presentation. The median time from the symptom onset to the presentation was 14 days (IQR 9, 21). At the time of the initial presentation, 45% (29 cases) had already received prior antibiotic therapy, with Penicillin or Penicillin combined with Beta-Lactamase inhibitors as the most commonly used agents.

### 3.3. Clinical Presentation (CP)

The classic triad of suppurative otitis media, unilateral facial pain, and ipsilateral abducens nerve palsy, indicative of complete Gradenigo syndrome, was observed in only 22% of cases (14 patients) at the initial presentation. Incomplete Gradenigo syndrome was present in 74% (48 patients), while the remaining 3% (2 patients) did not exhibit otorrhea, unilateral facial pain, or ipsilateral abducens palsy at the time of the initial presentation. Common additional symptoms included fever, otalgia, and a general decline in conditions.

**Figure 2:**
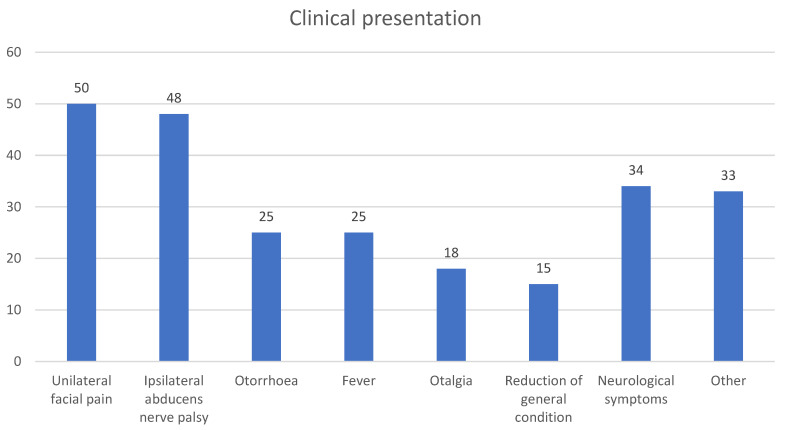
Clinical presentation of Gradenigo syndrome. Frequency of symptoms observed at the initial presentation.

Neurological symptoms were present in 52% (34 patients) at the time of the initial presentation, most frequently neck stiffness, photophobia, facial palsy, vomiting, and an altered mental state. Clinically suspected mastoiditis was observed in 11% (7 patients) at the time of presentation (Table 1, Figure 2).

Reported laboratory findings included C-reactive protein (reference value: 0–0.5 mg/dL), total leukocytes (reference value: 4000–17,500/µL), the erythrocyte sedimentation rate (ESR) (reference value: 0–10 mm/h), and neutrophil granulocytes (reference value: 50–70% of total leukocytes). CRP levels were reported in 38% of cases (25 patients), with a median value of 5 mg/dL. Total leukocyte counts were reported in 48% of cases (31 patients), with a median value of 12,200/µL. Neutrophil granulocytes were available in 31% of cases (20 patients), with a median percentage of 74, while the ESR was reported in 20% of cases (13 patients), with a median value of 67 mm/h.

### 3.4. Radiological Criteria (RC)

Initial imaging modalities included computed tomography (CT) in 82% of cases (53 patients) and magnetic resonance imaging (MRI) in 85% of cases (55 patients). Angiographic imaging was performed in 20% of cases (13 patients), while scintigraphy was used in only 3% (2 patients) (Table 2).

#### 3.4.1. CT

**Figure 3:**
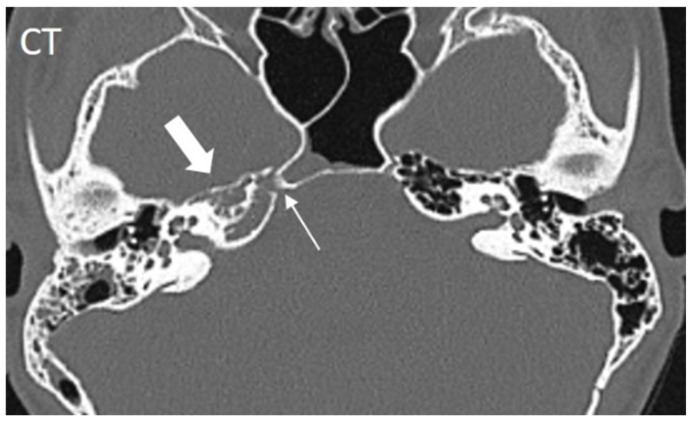
CT of a young patient with clinically present Gradenigo syndrome. It shows the lytic destruction of the bone trabeculae of the petrous apex as well as its obliteration without expansion (thick arrow). The course of the abducens nerve in the Dorello canal through the affected petrous apex can be visualized (thin arrow).

Mastoiditis was identified in 45% of cases, and the obliteration of the petrous bone tip was noted in 49% of cases (Figure 3). Other findings included the lytic destruction of the bone trabeculae and vascular involvement.

#### 3.4.2. MRI

**Figure 4:**
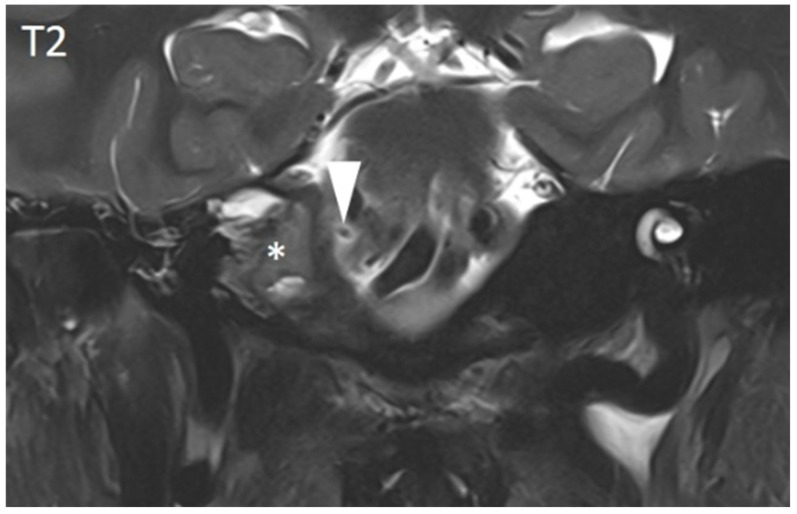
MRI (with contrast agent) of a young patient with clinically present Gradenigo syndrome. The image of an inflammatory pseudotumor in the petrous apex (star) correlates with the computed tomography images. The coronary T2 images allow the direct visualization of the abducens nerve (arrowhead) adjacent to the inflammatory changes.

Findings most frequently demonstrated inflammation in the petrous apex (Figure 4). The inflammatory involvement of the internal carotid artery and meningeal enhancement were each observed in 27% of cases.

### 3.5. Therapeutic Interventions (TIs)

Details regarding antibiotic therapy during hospitalization were provided in 57 patients (88%). A surgical intervention was reported in 47 patients (72%). Systemic glucocorticoid therapy was administered in 19 patients (31%), antiviral therapy was administered in 4 patients (7%), and anticoagulant medication was administered in 25% of cases.

#### 3.5.1. Antibiotic Treatment (AB)

Antibiotic therapy was categorized based on inpatient or outpatient administration (Table 3). Any adjustments to the therapy and the reasons for such changes were documented.

##### Inpatient Stay

**Figure 5:**
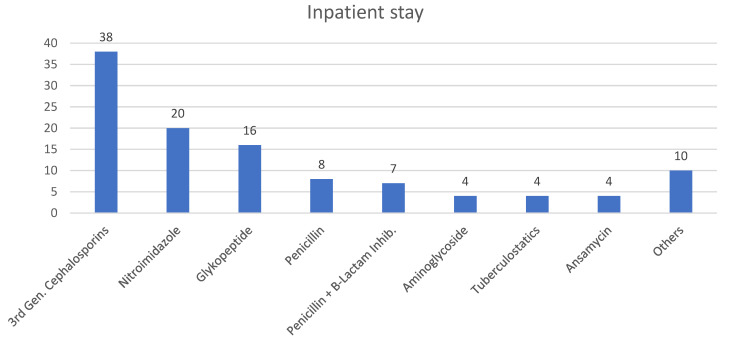
Antibiotics administered during inpatient stay.

Third-generation cephalosporins were the most frequently administered antibiotics, followed by nitroimidazoles and glycopeptides. The most common antibiotic combination therapy used in the inpatient setting consisted of third-generation cephalosporins and nitroimidazoles, administered in 10 cases (18%) (Figure 5). In seven cases (12%), a combination of third-generation cephalosporins, nitroimidazoles, and glycopeptides was administered, while in six cases (11%), nitroimidazole was omitted from this combination. Penicillin, with or without Beta-Lactamase inhibitors, was administered in 15 cases. Adjustments to the antibiotic regimen were made in 19 cases (33%) during the hospital stay, primarily due to clinical deterioration (47%) or to align with microbiological results (26%). The median duration of the inpatient antibiotic therapy, reported in 43 cases, was 21 days (IQR 10, 35).

##### Outpatient Stay

**Figure 6:**
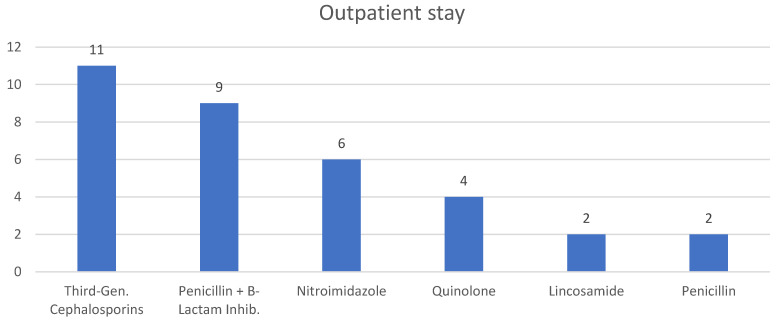
Antibiotics administered during outpatient stay.

Post-discharge antibiotic continuation was reported in 32 cases (49%). Oral administration was used in 21 cases (72%), while parenteral administration was utilized in 8 cases (28%). Similarly to inpatient therapy, third-generation cephalosporins were the most commonly prescribed antibiotics in outpatient therapy (Figure 6). The median duration of the outpatient antibiotic therapy, reported in 27 cases, was 26 days (IQR 14, 42).

#### 3.5.2. Surgical Therapy

Surgical intervention was documented in 47 patients (72%). Myringotomy was the most commonly performed procedure (36 cases/77%), followed by a subsequent ventilation tube insertion (33 cases/70%) and mastoidectomy (32 cases/68%). Direct surgical access to the petrous apex was not employed in any of the reported cases. In 19 patients (29%), surgical therapy consisting of a simultaneous mastoidectomy, paracentesis, and tympanostomy tube insertion was performed.

### 3.6. Microbiological Analysis (MA)

**Figure 7:**
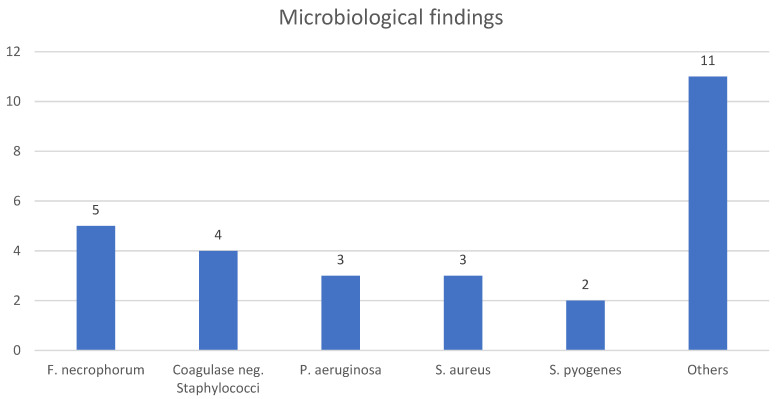
Frequency of detected bacterial pathogens.

A microbiological analysis was reported in 56 patients (86%). Bacterial pathogens were identified in 23 cases (41%). Pathogens were detected in blood cultures in four cases (17%) and in cerebrospinal fluid in two patients (9%). In surgical specimens or swabs, bacteria were identified in 20 patients (87%). The most frequently identified organisms were *Fusobacterium necrophorum*, *coagulase-negative staphylococci*, *Staphylococcus aureus*, *Pseudomonas aeruginosa*, and *Streptococcus pyogenes* (Table 4, Figure 7).

### 3.7. Outcome

Clinical outcomes were available for 64 of the 65 reported cases (98%). Clinical improvement was noted in 63 patients (98%), with a complete resolution of symptoms in 47 cases (73%). Complications related to treatment were reported in 18 patients (29%), and there was one reported fatality (2%). Notably, patients who presented with neurological symptoms at the time of the initial consultation appeared to have a higher likelihood of experiencing complications during treatment. The presence of the complete clinical triad of Gradenigo syndrome did not demonstrate a significant association with unfavorable clinical outcomes or higher complication rates compared to incomplete presentations (all *p* > 0.53). On average, clinical improvement was documented 11 days (IQR 7, 33) after the surgical intervention (e.g., mastoidectomy) and 14 days (IQR 7, 37) after the initiation of the inpatient antibiotic therapy.

## 4. Discussion

This systematic review presents a comprehensive overview of the incidence, diagnostic strategies, therapeutic interventions, and clinical outcomes of Gradenigo syndrome in the pediatric population—a rare but potentially life-threatening complication of acute otitis media. Gradenigo syndrome is characterized by the spread of the infection to the petrous apex, leading to the inflammatory impairment of the associated cranial nerves. This results in the classic triad of symptoms—otorrhea, unilateral facial pain, and horizontal diplopia—first described in the early 20th century [18].

The median age at diagnosis in our study cohort was 8 years (IQR 5.0, 11.5), which is slightly outside the peak incidence of acute otitis media in childhood described in the literature [86]. The gender distribution was balanced, with 48% male and 52% female patients.

The median time interval from the episode of acute otitis media to Gradenigo syndrome symptoms was 25 days (IQR 10, 30). This delay underscores the potential for late complications, even after the primary infection has ostensibly resolved, highlighting the importance of a thorough medical history in cases of suspected Gradenigo syndrome.

At the time of the initial presentation, only 22% of cases exhibited the classic symptom triad of Gradenigo syndrome. The majority (74%) presented with an incomplete constellation of symptoms, while 3% (n = 2) showed none of the typical features at the initial presentation. In one of the two cases, although no active otorrhea was present at the time of the presentation, a history of recurrent ear discharge was reported [27]. In the other case, abducens nerve palsy developed during the inpatient antibiotic therapy [82]. Beyond the classical signs, the most frequently reported additional symptoms included neurological manifestations (52%), fever (38%), otalgia (28%), and a general decline in the clinical condition (23%). Notably the presence of the complete clinical triad of Gradenigo syndrome did not demonstrate a significant association with unfavorable clinical outcomes or higher complication rates compared to incomplete presentations (all *p* > 0.53). These findings illustrate the diagnostic challenges associated with Gradenigo syndrome in children, particularly due to its highly variable and often nonspecific presentation. The underlying petrous apicitis may initially manifest without the full triad, necessitating a high index of clinical suspicion. Given the potential for overlapping or concomitant complications of otitis media—such as facial nerve palsy, labyrinthitis, or intracranial abscesses—these differential diagnoses should be actively considered. The need for adjunct diagnostic tools, including laboratory parameters and especially imaging modalities (e.g., CT or MRI of the temporal bone and skull base), is therefore evident and essential for an early and accurate diagnosis.

Radiological assessments primarily involved computed tomography (CT) (82%) and magnetic resonance imaging (MRI) (85%), with angiographic modalities used in 20% of cases to evaluate potential vascular involvement. CT imaging typically revealed secondary inflammatory changes in the bony structures extending to the petrous bone, while MRI was more effective in detecting the direct inflammatory involvement of the petrous apex and associated cranial nerves. Both modalities are capable of visualizing vascular involvement. CT is particularly valuable for surgical planning due to its detailed visualization of bony structures, whereas MRI provides superior insight into the inflammatory process and disease progression. When both imaging modalities are available, a combined approach appears to be the most appropriate for optimal diagnostic accuracy and treatment planning. Given the absence of radiation exposure, MRI is particularly well suited for follow-up imaging in children, allowing for the accurate monitoring of the extent of the inflammation and effectiveness of the therapy over time.

The microbiological analysis revealed a diverse array of pathogens, including both aerobic and anaerobic bacteria. Commonly isolated organisms included Fusobacterium necrophorum (Gram-negative, obligate anaerobic), coagulase-negative staphylococci (Gram-positive, facultative anaerobic), Pseudomonas aeruginosa (Gram-negative, obligate aerobic), and Staphylococcus aureus (Gram-positive, facultative anaerobic), reflecting the polymicrobial nature of the infection. Given the proximity to critical cranial structures and the frequent occurrence of neurological symptoms at presentation, empiric antibiotic therapy should provide coverage against both aerobic and anaerobic pathogens and ensure the adequate penetration into the central nervous system (CNS). In our cohort, the inpatient administration of third-generation cephalosporins was the only regimen associated with a significantly reduced complication rate (*p* = 0.016). Accordingly, a combination of a third-generation cephalosporin with a nitroimidazole, as most commonly employed in the reviewed cases, appears to fulfill the above requirements and constitutes a rational empiric treatment approach. Once microbiological results are available, therapy can be de-escalated to a targeted regimen, which may be continued on an outpatient basis. However, due to the limited number of cases and the heterogeneity of the data, significant differences in outcomes based on specific antibiotic regimens for the outpatient setting could not be determined.

If conservative treatment fails or complications develop in Gradenigo syndrome, surgical intervention becomes necessary. In the cohort studied, surgical procedures were performed in 72% of cases. Myringotomy was the most frequently performed procedure, accounting for 77%, followed by the insertion of ventilation tubes in 70% and mastoidectomy in 68%. Due to the limited sample size, no significant differences in outcomes could be determined based on the type of surgical intervention.

Clinical improvement, defined as the recovery of abducens nerve function as assessed by an ophthalmologic examination, was observed in 98% of cases. The improvement occurred at a median of 14 days (IQR 7, 37) after initiating the antibiotic therapy and 11 days (IQR 7, 33) following the surgical intervention. This timeline provides clinicians with valuable insight into treatment responses and helps prevent unnecessary therapeutic escalation. While this study lacked sufficient power to detect significant differences in outcomes between conservative and surgical treatments, the overall high rate of symptom resolution (73%) supports the multimodal therapeutic approach commonly employed. Therapy-related complications occurred in 29% of cases, most commonly involving recurrent symptoms following the transition from parenteral to oral antibiotics, underscoring the importance of the adequate duration of the intravenous therapy. Patients who presented with neurological complications at the time of the initial evaluation also appeared to have a higher complication rate, underscoring the importance of the early initiation of treatment. A single fatality (2%) was reported.

## 5. Conclusions

Gradenigo syndrome is a rare but potentially life-threatening complication of bacterial otitis media in children. Its variable and often incomplete clinical presentation, along with the absence of a consistent causative pathogen, underscores the importance of comprehensive diagnostic imaging. A combination of CT and MRI provide complementary information for diagnosis, with MRI preferred for follow-up in children due to its lack of radiation exposure.

Broad-spectrum antibiotics remain the cornerstone of therapy, while surgical intervention is frequently required in more severe or refractory cases. Following the initial clinical improvement, the continuation of targeted long-term antibiotic therapy is recommended to ensure a complete resolution and prevent recurrence.

Despite high rates of clinical improvement, the substantial incidence of treatment-related complications underscores the need for individualized management strategies.

## 6. Limitations

This scoping review is limited by the nature of the available literature, primarily consisting of single case reports or small case series. In some cases, the data provided, particularly laboratory findings and long-term outcomes, was incomplete. The inclusion of only English, German, and French reports may have introduced a selection bias. Furthermore, this scoping review is limited by the rarity of Gradenigo syndrome and the consequently small number of reported pediatric cases, which restricts the ability to draw robust causal inferences.

## Figures and Tables

**Table 1:** Clinical presentation of Gradenigo syndrome. Frequency of symptoms observed at the initial presentation, including classic and incomplete triad components.

CP	Clinical Symptom	N/%	Neurological Symptom	N/%
	Unilateral facial pain	50/77		
	Ipsilateral abducens nerve palsy	48/74		
	Otorrhea	25/38		
	Fever	25/38		
	Otalgia	18/28		
	Reduction in general condition	15/23		
	Other	33/51		
	Neurological symptoms	34/52	Neck stiffness	11/32
			Vomiting	10/29
			Photophobia	7/21
			Altered mental state	6/18
			Facial palsy	5/15
			Headache	4/12
			Vertigo	2/6
			Dysphasia	1/3
			Ptosis	1/3

**Table 2:** Radiological findings in Gradenigo syndrome. Summary of CT and MRI findings, including the prevalence of mastoiditis, petrous bone obliteration, and other significant radiological features.

Modality	RC	N/%
*CT*	Obliteration of the petrous bone tip	26/49
	Vascular involvement	25/47
	Mastoiditis	24/45
	Lytic destruction of the bone trabeculae	14/26
	Sinus thrombosis	3/6
	Prominent pneumatization of the petrous bone tip	2/4
	Narrowing of the internal carotid artery	1/2
	Other	21/40
*MRT*	Inflammatory process in the petrous apex	46/84
	Enhancement of the meninges	15/27
	Inflammatory involvement of the internal carotid artery	15/27
	Sinus thrombosis	8/15
	Cerebral nerve enhancement	5/9
	Other	17/31

**Table 3:** Antibiotic therapy during inpatient and outpatient care. Details on the types and frequencies of antibiotics administered during hospital stay and after discharge.

TI	Setting	AB	N/%
	*Inpatient stay*	3rd Gen. Cephalosporins	38/67
		Nitroimidazole	20/35
		Glykopeptide	16/28
		Penicillin	8/14
		Penicillin + B-Lactam Inhib.	7/12
		Aminoglycoside	4/7
		Tuberculostatics	4/7
		Ansamycin	4/7
		Epoxide	3/5
		Carbapenem	2/4
		Lincosamide	2/4
		2nd Gen. Cephalosporins	1/2
		4th Gen. Cephalosporins	1/2
		Macrolide	1/2
	*Outpatient stay*	3rd Gen. Cephalosporins	11/37
		Penicillin + B-Lactam Inhib.	9/30
		Nitroimidazole	6/20
		Quinolone	4/13
		Lincosamide	2/7
		Penicillin	2/7
		Epoxide	1/3
		Oxazolidinone	1/3
		Glykopeptide	1/3
		2nd Gen. Cephalosporins	1/3
		Cephalosporins unspecified	1/3

**Table 4:** Microbiological findings. Summary of microbiological results, including types of bacteria detected and their frequencies.

MA	Organism	N/%
*Microbiological finding*	*F. necrophorum*	5/22
	*Coagulase neg. Staphylococci*	4/17
	*P. aeruginosa*	3/13
	*S. aureus*	3/13
	*S. pyogenes*	2/9
	*S. acidominimus*	1/4
	*S. intermedius*	1/4
	*S. capitis*	1/4
	*S. pneumoniae*	1/4
	*S. constellatus*	1/4
	*E. coli*	1/4
	*Diphteroids*	1/4
	*M. tuberculosis*	1/4
	*S. viridans*	1/4
	*Enterococcus*	1/4
	*Proteus*	1/4

## References

[1] Kaur R., Morris M., Pichichero M.E. (2017). Epidemiology of Acute Otitis Media in the Postpneumococcal Conjugate Vaccine Era. Pediatrics.

[2] Leung A.K.C., Wong A.H.C. (2017). Acute Otitis Media in Children. Recent Pat. Inflamm. Allergy Drug Discov..

[3] El Feghaly R.E., Nedved A., Katz S.E., Frost H.M. (2023). New insights into the treatment of acute otitis media. Expert Rev. Anti-Infect. Ther. Ther..

[4] Gaddey H.L., Wright M.T., Nelson T.N. (2019). Otitis Media: Rapid Evidence Review. Am. Fam. Physician.

[5] Meherali S., Hartling L., Campbell A., Robin F., Scott S. (2021). Parent information needs and experience regarding acute otitis media in children: A systematic review. Patient Educ. Couns..

[6] Suzuki H.G., Dewez J.E., Nijman R.G., Yeung S. (2020). Clinical practice guidelines for acute otitis media in children: A systematic review and appraisal of European national guidelines. BMJ Open.

[7] Venekamp R.P., Sanders S.L., Glasziou P.P., Del Mar C.B., Rovers M.M. (2015). Antibiotics for acute otitis media in children. Cochrane Database Syst. Rev..

[8] Holm N.H., Rusan M., Ovesen T. (2020). Acute otitis media and antibiotics—A systematic review. Dan. Med. J..

[9] Hutz M.J., Moore D.M., Hotaling A.J. (2018). Neurological Complications of Acute and Chronic Otitis Media. Curr. Neurol. Neurosci. Rep..

[10] Smolinski N.E., Djabali E.J., Al-Bahou J., Pomputius A., Antonelli P.J., Winterstein A.G. (2024). Antibiotic treatment to prevent pediatric acute otitis media infectious complications: A meta-analysis. PLoS ONE.

[11] Cushen R., Francis N.A. (2020). Antibiotic use and serious complications following acute otitis media and acute sinusitis: A retrospective cohort study. Br. J. Gen. Pract..

[12] Juilland N., Vinckenbosch P., Richard C. (2016). Acute otitis media and short-term complications. Rev. Med. Suisse..

[13] Schilder A.G., Chonmaitree T., Cripps A.W., Rosenfeld R.M., Casselbrant M.L., Haggard M.P., Venekamp R.P. (2016). Otitis media. Nat. Rev. Dis. Primers.

[14] Bhutta M.F., Monono M.E., Johnson W.D. (2020). Management of infective complications of otitis media in resource-constrained settings. Curr. Opin. Otolaryngol. Head Neck Surg..

[15] Loh R., Phua M., Shaw C.L. (2018). Management of paediatric acute mastoiditis: Systematic review. J. Laryngol. Otol..

[16] Lavin J.M., Rusher T., Shah R.K. (2016). Complications of Pediatric Otitis Media. Otolaryngol. Head Neck Surg..

[17] Favre N., Patel V.A., Carr M.M. (2021). Complications in Pediatric Acute Mastoiditis: HCUP KID Analysis. Otolaryngol. Head Neck Surg..

[18] Gradenigo G. (1907). Über die Paralyse des Nervus abducens bei Otitis. Archiv für Ohrenheilkunde.

[19] Sousa Menezes A., Ribeiro D., Balona F., Maré R., Azevedo C., Rocha J., Dias L. (2020). Gradenigo’s Syndrome with Carotid Septic Stenosis. Case Rep. Otolaryngol..

[20] Heshin-Bekenstein M., Megged O., Peleg U., Shahroor-Karni S., Bass R., Benifla M., Bar-Meir M. (2014). Gradenigo’s syndrome: Is fusobacterium different? Two cases and review of the literature. Int. J. Pediatr. Otorhinolaryngol..

[21] Rifai Y., Cassimatis N., Soliman Md I. (2023). Neurosurgical Intervention in the Treatment of Gradenigo Syndrome: A Case Report. Cureus.

[22] McLaren J., Cohen M.S., El Saleeby C.M. (2020). How well do we know Gradenigo? A comprehensive literature review and proposal for novel diagnostic categories of Gradenigo’s syndrome. Int. J. Pediatr. Otorhinolaryngol..

[23] Talmor G., Vakil M., Tseng C., Svider P., Ying M., Eloy J.A. (2022). Petrous Apicitis: A Systematic Review and Case Presentation. Otol. Neurotol..

[24] Gadre A.K., Chole R.A. (2018). The changing face of petrous apicitis-a 40-year experience. Laryngoscope.

[25] R Core Team (2024). R: A Language and Environment for Statistical Computing.

[26] Piron K., Gordts F., Herzeel R. (2003). Gradenigo syndrome: A case-report. Bull. Soc. Belg. Ophtalmol..

[27] Cundiff J.G., Djalilian H.R., Mafee M.F. (2006). Bilateral sequential petrous apicitis secondary to an anaerobic bacterium. Otolaryngol. Head Neck Surg..

[28] Jacobsen C.L., Bruhn M.A., Yavarian Y., Gaihede M.L. (2012). Mastoiditis and Gradenigo’s Syndrome with anaerobic bacteria. BMC Ear Nose Throat Disord..

[29] Uchoa J., de Carvalho Leal M., Lima Medeiros D., Sousa Reis M., de Lavor Monteiro V.A., Farias Barbosa M.E., Araújo Lessa M.D. (2022). Gradenigo Syndrome as a rare complication of otitis: A case report. Int. Arch. Otorhinolaryngol..

[30] Al-Ammar A.Y. (2001). Recurrent temporal petrositis. J. Laryngol. Otol..

[31] Burston B.J., Pretorius P.M., Ramsden J.D. (2005). Gradenigo’s syndrome: Successful conservative treatment in adult and paediatric patients. J. Laryngol. Otol..

[32] Choi K.Y., Park S.K. (2014). Petrositis With Bilateral Abducens Nerve Palsies complicated by Acute Otitis Media. Clin. Exp. Otorhinolaryngol..

[33] Ferguson P. (2016). Gradenigo syndrome: A rare complication of otitis media. Marshall J. Med..

[34] Finkelstein Y., Marcus N., Mosseri R., Bar-Sever Z., Garty B.Z. (2003). Streptococcus acidominimus infection in a child causing Gradenigo syndrome. Int. J. Pediatr. Otorhinolaryngol..

[35] Ghani S., Likeman M., Lyttle M.D. (2017). New onset strabismus in association with ear pain. Arch. Dis. Child. Educ. Pract. Ed..

[36] Humayun H.N., Akhtar S., Ahmed S. (2011). Gradenigo’s syndrome--surgical management in a child. J. Pak. Med. Assoc..

[37] Janjua N., Bajalan M., Potter S., Whitney A., Sipaul F. (2016). Multidisciplinary care of a paediatric patient with Gradenigo’s syndrome. BMJ Case Rep..

[38] Jensen P.V.F., Avnstorp M.B., Dzongodza T., Chidziva C., von Buchwald C. (2017). A fatal case of Gradenigo’s syndrome in Zimbabwe and the Danish-Zimbabwean ENT collaboration. Int. J. Pediatr. Otorhinolaryngol..

[39] Shiffali P., Sandhu K.S., Singh J. (2017). Rare case of chronic suppurative otitis media with gradenigo syndrome. Int. J. Otorhinolaryngol. Head Neck Surg..

[40] Khurshid R.S., MIaSIaRA (2019). Gradenigo’s Syndrome: A Report of Two Cases with Review of Literature. Internet J. Otorhinolaryngol..

[41] Kong S.K., Lee I.W., Goh E.K., Park S.E. (2011). Acute otitis media-induced petrous apicitis presenting as the Gradenigo syndrome: Successfully treated by ventilation tube insertion. Am. J. Otolaryngol..

[42] Koral K., Dowling M. (2006). Petrous apicitis in a child: Computed tomography and magnetic resonance imaging findings. Clin. Imaging.

[43] Marianowski R., Rocton S., Ait-Amer J.L., Morisseau-Durand M.P., Manach Y. (2001). Conservative management of Gradenigo syndrome in a child. Int. J. Pediatr. Otorhinolaryngol..

[44] Marteau E., Georget-Bouquinet E., Verlhac S., Gauthier A., Remus N., Madhi F. (2011). Successful prolonged conservative treatment of Gradenigo’s syndrome in a 4-year-old girl: A case report and literature review. Int. J. Pediatr. Otorhinolaryngol. Extra.

[45] Rossor T.E., Anderson Y.C., Steventon N.B., Voss L.M. (2011). Conservative management of Gradenigo’s syndrome in a child. BMJ Case Rep..

[46] Silva Neto A.R., Bezerra M.J., Galvão A.R., Rocha T.A., Pereira M.G., Ferreira V.V. (2009). Gradenigo Syndrome: Case Report and Review of Literature. Neurobiologia.

[47] Vitale M., Amrit M., Arora R., Lata J. (2017). Gradenigo’s syndrome: A common infection with uncommon consequences. Am. J. Emerg. Med..

[48] Yoong H.S., Kiaang T.K. (2006). Gradenigo’s syndrome presenting as a tumor. Otolaryngol. Head Neck Surg..

[49] Bloching M., Heider C., Amm S., Kösling S. (2005). Gradenigo syndrome--still a threatening complication of otitis media. HNO.

[50] Favier M., Bessou P., Franco-Vidal V., Pédespan J.M. (2015). Gradenigo syndrome and petrositis in a child. Arch. Pediatr..

[51] Gibier L., Darrouzet V., Franco-Vidal V. (2009). Gradenigo syndrome without acute otitis media. Pediatr. Neurol..

[52] Ibrahim M., Shah G., Parmar H. (2010). Diffusion-weighted MRI identifies petrous apex abscess in Gradenigo syndrome. J. Neuroophthalmol..

[53] Karunakaran T., Kaneshamoorthy M., Harris R. (2016). Clivus erosions following Gradenigo’s syndrome-mastoiditis causing VI nerve palsy. BMJ Case Rep..

[54] Nikkhah A. (2014). Gradenigo’s Syndrome Without Facial Nerve Involvement in a Fourteen-Month-Old Girl. Jentashapir J. Health Res..

[55] Price T., Fayad G. (2002). Abducens nerve palsy as the sole presenting symptom of petrous apicitis. J. Laryngol. Otol..

[56] Zengel P., Wiekström M., Jäger L., Matthias C. (2007). Isolated apical petrositis: An atypical case of Gradenigo’s syndrome. HNO.

[57] Rossi N., Swonke M.L., Reichert L., Young D. (2019). Gradenigo’s syndrome in a four-year-old patient: A rare diagnosis in the modern antibiotic era. J. Laryngol. Otol..

[58] Al-Faifi J.A. (2023). Gradenigo Syndrome in a 6-Year-Old Boy with Acute Otitis Media: A Case Report. Int. Tinnitus J..

[59] Bozan N., Düzenli U., Yalinkilic A., Ayral A., Parlak M., Turan M., Kiroglu A.F. (2018). Gradenigo Syndrome Induced by Suppurative Otitis Media. J. Craniofacial Surg..

[60] Dorner R.A., Ryan E., Carter J.M., Fajardo M., Marsden L., Fricchione M., Higgins A. (2017). Gradenigo Syndrome and Cavitary Lung Lesions in a 5-Year-Old With Recurrent Otitis Media. J. Pediatr. Infect. Dis. Soc..

[61] De Foer B., Vercruysse J.P., Spaepen M., Somers T., Pouillon M., Offeciers E., Casselman J.W. (2010). Diffusion-weighted magnetic resonance imaging of the temporal bone. Neuroradiology.

[62] Villa G., Lattere M., Rossi A., Di Pietro P. (2005). Acute onset of abducens nerve palsy in a child with prior history of otitis media: A misleading sign of Gradenigo syndrome. Brain Dev..

[63] Sethi A., Sabherwal A., Gulati A., Sareen D. (2005). Primary tuberculous petrositis. Acta Otolaryngol..

[64] Park S.N., Yeo S.W., Suh B.D. (2003). Cavernous sinus thrombophlebitis secondary to petrous apicitis: A case report. Otolaryngol. Head Neck Surg..

[65] Colpaert C., Van Rompaey V., Vanderveken O., Venstermans C., Boudewyns A., Menovsky T., de Veuster I., Van de Heyning P., Hamans E. (2013). Intracranial complications of acute otitis media and Gradenigo’s syndrome. B-ENT.

[66] Trimis G., Mostrou G., Lourida A., Prodromou F., Syriopoulou V., Theodoridou M. (2003). Petrositis and cerebellar abscess complicating chronic otitis media. J. Paediatr. Child. Health.

[67] Sethi A., Sethi D., Mrig S., Passey J.C., Srivastav N. (2006). Coexistent acute pyogenic and tubercular petrous apicitis: A diagnostic dilemma. J. Laryngol. Otol..

[68] Athapathu A.S., Bandara E.R.S., Aruppala A., Chandrapala K., Mettananda S. (2019). A child with Gradenigo syndrome presenting with meningism: A case report. BMC Pediatr..

[69] Özkaçmaz S. (2019). Acute otitis media associated with Gradenigo syndrome transverse sinus thrombosis: A case report. J. Int. Med. Res..

[70] Mendes C., Garrido C., Guedes M., Marques L. (2014). Gradenigo syndrome: An unexpected otitis complication. Nascer E Crescer.

[71] Chan K.C., Chen S.L. (2023). Diplopia in a Child: Gradenigo Syndrome Is an Unforgettable Disease. Ear Nose Throat J..

[72] Bonavia L., Jackson J. (2022). Gradenigo Syndrome in a 14-Year-old Girl as a Consequence of Otitis Media With Effusion. J. Neuroophthalmol..

[73] Costa J.V., João M., Guimarães S. (2020). Bilateral papilledema and abducens nerve palsy following cerebral venous sinus thrombosis due to Gradenigo’s syndrome in a pediatric patient. Am. J. Ophthalmol. Case Rep..

[74] Demir B., Abuzaid G., Ergenc Z., Kepenekli E. (2020). Delayed diagnosed Gradenigo’s syndrome associated with acute otitis media. SAGE Open Med. Case Rep..

[75] Rho Y.-I. (2015). Headache Attributed to Petrous Apicitis without Symptoms of Acute Otitis Media. Austin. J. Clin. Neurol..

[76] Joana P.B., Cristina A., Aníbal C. (2022). Gradenigo’s syndrome—A Diagnostic Challenge. J. Pediatr. Neonatol..

[77] Scardapane A., Del Torto M., Nozzi M., Elio C., Breda L., Chiarelli F. (2010). Gradenigo’s syndrome with lateral venous sinus thrombosis: Successful conservative treatment. Eur. J. Pediatr..

[78] Savasta S., Canzi P., Aprile F., Michev A., Foiadelli T., Manfrin M., Benazzo M. (2019). Gradenigo’s syndrome with abscess of the petrous apex in pediatric patients: What is the best treatment?. Child’s Nerv. Syst..

[79] Guarnizo A., Rugilo C. (2023). Gradenigo’s syndrome associated to internal carotid artery vasculitis. Acta Neurol. Belg..

[80] Brambilla A., Pasti M., Parri N. (2019). Sudden Diplopia at a Pediatric Emergency Department: A Case of Gradenigo Syndrome in a Child. Pediatr. Emerg. Care.

[81] Lutter S.A., Kerschner J.E., Chusid M.J. (2005). Gradenigo syndrome: A rare but serious complication of otitis media. Pediatr. Emerg. Care.

[82] Solms J., Evangelista M., Gourishankar A. (2017). A Child With Right Ear Pain and a Gaze Palsy. Clin. Pediatr..

[83] Jensen P.V., Hansen M.S., Møller M.N., Saunte J.P. (2016). The Forgotten Syndrome? Four Cases of Gradenigo’s Syndrome and a Review of the Literature. Strabismus.

[84] Jacob A., Alsarhan A., Nazir T., Kherani S., Bitar M.A. (2022). Beyond Gradenigo syndrome: Facial palsy and cavernous sinus involvement in a young teenage girl. Otolaryngol. Case Rep..

[85] Visosky A.M., Isaacson B., Oghalai J.S. (2006). Circumferential petrosectomy for petrous apicitis and cranial base osteomyelitis. Otol. Neurotol..

[86] Daly K.A., Giebink G.S. (2000). Clinical epidemiology of otitis media. Pediatr. Infect. Dis. J..

